# Housing and Environmental Uncertainty in Constructions of Exclusion Arising From Critical Life-Course Ruptures

**DOI:** 10.1093/geroni/igaa057.2004

**Published:** 2020-12-16

**Authors:** Kieran Walsh, Anna Urbaniak, Bridin Carroll

**Affiliations:** 1 Irish Centre for Social Gerontology, National University of Ireland Galway, Galway, Galway, Ireland; 2 NUI Galway, Galway, Galway, Ireland

## Abstract

There is growing recognition that the older adult life course can involve critical transitions that function as significant sources of adversity, and ruptures in life trajectories. While knowledge about how these ruptures generate multidimensional disadvantage remains underdeveloped, less is known about how they are spatially constituted and how their processes and outcomes may be mediated by older peoples’ relationship with place. Utilizing a ‘sense of home’ as a conceptual orientation, this paper explores the role of place in social exclusion arising from life-course ruptures. Focusing on bereavement, dementia on-set and forced migration, it draws data from 45 life-course interviews. Place (e.g. home environment and the wider community) was involved in three ways: as a component of the rupture; as a life domain where people experience exclusion; and as a mediator of exclusionary processes. Circularity is observed, with perceived environmental uncertainty intensifying effects of rupture-related exclusion, further contributing to that uncertainty.

